# 

**Published:** 1893-08-19

**Authors:** 


					5 BOSPTTA r 1 August 19th* '
wiUAL. WW . ' WITH EXTRA NURSING SUPPLEMENT
U PRICE TWOPENCE. ll if IP Per Annum, 8s. 6d? post free.
Jo. 360, Vol. XVI. Aug. 19th, 1893. m JETE> Monthly Parts, ?d.; post free, 8d.
'Wistered at the General Post Office m a Newspaper for ^ Ditto per Annum, 8*., post free,
transmission in the United Kingdom and Abroad.]
No. 360. Yol. XIV. Saturday,
A.tjgust 19th, 1893.
[Twopence.

				

